# Environmental Free-Living Amoebae Isolated from Soil in Khon Kaen, Thailand, Antagonize *Burkholderia pseudomallei*

**DOI:** 10.1371/journal.pone.0167355

**Published:** 2016-11-29

**Authors:** Parumon Noinarin, Pisit Chareonsudjai, Pinich Wangsomnuk, Surasak Wongratanacheewin, Sorujsiri Chareonsudjai

**Affiliations:** 1 Department of Microbiology, Faculty of Medicine, Khon Kaen University, Khon Kaen, Thailand; 2 Department of Environmental Science, Faculty of Science, Khon Kaen University, Khon Kaen, Thailand; 3 Department of Biology, Faculty of Science, Khon Kaen University, Khon Kaen, Thailand; 4 Melioidosis Research Center, Khon Kaen University, Khon Kaen, Thailand; 5 Biofilm Research Group, Khon Kaen University, Khon Kaen, Thailand; J Craig Venter Institute, UNITED STATES

## Abstract

Presence of *Burkholderia pseudomallei* in soil and water is correlated with endemicity of melioidosis in Southeast Asia and northern Australia. Several biological and physico-chemical factors have been shown to influence persistence of *B*. *pseudomallei* in the environment of endemic areas. This study was the first to evaluate the interaction of *B*. *pseudomallei* with soil amoebae isolated from *B*. *pseudomallei*-positive soil site in Khon Kaen, Thailand. Four species of amoebae, *Paravahlkampfia ustiana*, *Acanthamoeba* sp., *Naegleria pagei*, and isolate A-ST39-E1, were isolated, cultured and identified based on morphology, movement and 18S rRNA gene sequence. Co-cultivation combined with a kanamycin-protection assay of *B*. *pseudomallei* with these amoebae at MOI 20 at 30°C were evaluated during 0–6 h using the plate count technique on Ashdown’s agar. The fate of intracellular *B*. *pseudomallei* in these amoebae was also monitored by confocal laser scanning microscopy (CLSM) observation of the CellTracker^™^ Orange-*B*. *pseudomallei* stained cells. The results demonstrated the ability of *P*. *ustiana*, *Acanthamoeba* sp. and isolate A-ST39-E1 to graze *B*. *pseudomallei*. However, the number of internalized *B*. *pseudomallei* substantially decreased and the bacterial cells disappeared during the observation period, suggesting they had been digested. We found that *B*. *pseudomallei* promoted the growth of *Acanthamoeba* sp. and isolate A-ST39-E1 in co-cultures at MOI 100 at 30°C, 24 h. These findings indicated that *P*. *ustiana*, *Acanthamoeba* sp. and isolate A-ST39-E1 may prey upon *B*. *pseudomallei* rather than representing potential environmental reservoirs in which the bacteria can persist.

## Introduction

The soil-dwelling bacterium, *Burkholderia pseudomallei*, is the causative agent of a fatal infectious disease, melioidosis that is endemic particularly in Southeast Asia and northern Australia [1–3]. Physico-chemical parameters that facilitate persistence of *B*. *pseudomallei* in the environment include slightly acidic soil, a moisture content >10%, and relatively high chemical oxygen demand and total nitrogen [4, 5]. Moreover, soil microcosms with low salinity and iron were found to alter the bacterial morphology from a rod-like to a coccoid form, suggesting that such conditions are advantageous for its persistence in the environment and may increase the risk of transmission to humans [6]. Several biological factors have also been demonstrated to influence the survival of *B*. *pseudomallei*. Kaestli and colleagues noted its presence in native and exotic grasses in northern Australia, suggesting a potential for spread of *B*. *pseudomallei* by grazing animals [7]. In addition, the association of *B*. *pseudomallei* with germinating spores of the arbuscular mycorrhizal fungus *Gigaspora decipiens* emphasized the ability of the bacterium to interact with various eukaryotic cells [8]. Negative interactions with a closely related species, *Burkholderia ubonensis* in melioidosis-endemic areas have been demonstrated. The inactivation was caused by a pepsin-sensitive peptide moiety consistent with a bacteriocin-like compound, suggesting the application for biocontrol of this pathogen [9]. Another related species, *B*. *multivorans*, also antagonizes the growth of *B*. *pseudomallei* in soil [10]. The presence of *B*. *pseudomallei* in an agricultural crop soil was inversely related to the presence of antagonistic strains that can survive in a broader range of pH, temperatures and salt concentrations.

Free-living amoebae are also known to have diverse interactions with environmental bacteria. Such amoebae use bacteria as food sources and may be therefore considered to control microbial communities [11]. However, they may also act as “Trojan horses”, providing shelter and leading to long-term intra-amoeba survival of bacteria, thereby aiding bacterial survival and dispersal [12, 13]. Strategies to prevent engulfment and survive, or to replicate within protozoa, could have evolved among bacteria [11]. This phenomenon offers not only a protective reservoir but could also select for virulence behaviors that allow intracellular growth of bacteria, facilitating the transmission of infectious bacteria from the environment to humans [14]. Amoebae in the genera *Acanthamoeba*, *Dictyostelium*, *Hartmannella* and *Naegleria* can act as reservoirs for pathogenic bacteria [15, 16]. *Legionella pneumophila* is the most acknowledged intracellular bacterium harbored within free-living amoebae and has evolved mechanisms for survival in eukaryotic host cells [14]. Meanwhile, the endocytosis of *B*. *pseudomallei* into free-living amoebae belonging to the genus *Acanthamoeba* recovered from water samples in Australia suggested the possibility of environmental survival and subsequent human exposure [17]. Moreover, *B*. *pickettii* [18], *Campylobacter jejuni* [19], *Escherichia coli* [20, 21], *Helicobacter pylori* [22], *Listeria monocytogenes* [23], *Mycobacterium leprae* [24], *Shigella dysenteriae* and *S*. *sonnei* [25] and *Vibrio cholera* [26] have all been shown to have interactions with amoebae. However, not all these interactions are favorable for the bacterium since *M*. *bovis* was reported to be inactivated by environmental amoebae [27].

To the best of our knowledge, there is only limited information concerning the nature of any interactions between free-living amoebae isolated from soil and *B*. *pseudomallei*. The aim of our study was to investigate such interactions using free-living amoebae and *B*. *pseudomallei* isolated from the same soil site in Khon Kaen Province, Thailand. Isolated amoebae were cultured and maintained in the laboratory with living *Escherichia coli*. Based on morphology and 18S rRNA gene sequences, at least four species of amoebae were maintained and identified as *Paravahlkampfia ustiana*, *Acanthamoeba* sp., *Naegleria pagei* and isolate A-ST39-E1. A co-cultivation technique, combined with fluorescence staining and plate-count techniques, revealed a negative impact of these amoebae on *B*. *pseudomallei*.

## Materials and Methods

### Amoeba isolation and cultivation

Soil amoebae were isolated from a *B*. *pseudomallei-*positive soil site, site 39, [28] based on an enrichment method with slight modification [29] with the permission of the owner of the land. Briefly, 2 g of soil was placed at the center of a non-nutrient agar (1.5% agar) plate. Then 2 mL of a 0.03% tryptic soy broth (TSB) was gently dropped on the soil and incubated at 30°C (represent the average temperature of Khon Kaen province, Thailand) for 2 days in the dark, tightly wrapped to keep the humidity high. Soil amoebae were then detached from the agar surface by cooling the plate to 4°C for at least 3 min and flushing with 0.03% TSB. After leaving the sediment to settle for 3 min, the upper layer of the supernatant containing amoebae was transferred and diluted with 0.03% TSB for a limiting dilution in a 96-well tissue culture plate (Costar, Corning, NY, USA) and incubated at 30°C in darkness. Daily observation under an inverted microscope (Nikon Eclipse TS100, Japan) was used to look for wells containing single amoebic morphotypes. A single amoebic cell in each well was maintained by daily replacement with fresh 0.03% TSB daily until cells were 40–50% confluent. The amoebic cultures were thereafter maintained under monoxenic conditions with *E*. *coli* SM-10 as a food source in 24-well plates (Costar, Corning, NY, USA) at 30°C until cells were approximately 70% confluent. Trophozoites were gently washed 3 times with 100 μL of Page’s Amoebic Saline (PAS) (0.012% NaCl, 0.0004% MgSO_4_·7H_2_O, 0.0004% CaCl_2_·2H_2_O, 0.0142% Na_2_HPO_4_ and 0.0136% KH_2_PO_4_) [30] to remove most nutrients and resuspended in 4°C-PAS before enumeration using a hemocytometer.

For the co-cultivation experiment with the kanamycin-protection assay, amoeba trophozoites were pre-treated with a gradually increasing series of kanamycin concentrations (30–300 μg/mL) by changing PAS with kanamycin daily for 10 days. The treated amoeba trophozoites were therefore made tolerant to 300 μg/mL kanamycin.

### Morphological analysis

#### Bright-field microscopy

Amoebae (approximately 100 cells/10 μL PAS) were fixed with 10 μL of 2.5% (v/v) glutaraldehyde (EM grade; Electron Microscopy Sciences, Hatfield, PA) for 15 min and stained with either 10 μL of 0.4% (w/v) trypan blue or 0.1% (w/v) crystal violet for 30 sec. After 5 washes with PAS, the amoebae were post-fixed with 10 μL of 1.25% (v/v) glutaraldehyde and examined under a bright field microscope (Nikon, Eclipse Ni, Japan) at 1000× magnification. Examination of cyst morphology was performed after a culture had been left in PAS at room temperature for 7 days in a tightly closed microtube to allow starvation and oxygen limitation. The images were processed using the Axio Vision software.

#### DNA extraction and PCR

Genomic DNA was extracted from each amoeba culture using a QIAamp DNA Mini Kit (Qiagen, Germany). The 18S rRNA gene was amplified by PCR using 5 pairs of specific primers (Table 1) to achieve coverage of the full length of the gene. Each PCR reaction contained 30 ng of template DNA, 10 mM of each primer, 1.25 units of Taq DNA polymerase (RBC, Bioscience, Taipei, Taiwan), 10× Taq buffer with 15 mM MgCl_2_, 100 μM of each deoxynucleotide in a total volume of 25 μL. All PCR reactions used the same cycling conditions: incubation at 95°C for 5 min, followed by 35 cycles of 95°C for 30 seconds, 52°C for 30 sec and 72°C for 40 sec and a final extension at 72°C for 6 min (ABI thermocycler, Applied Biosystems, USA). Thereafter, the PCR products were gel-purified using the HiYield gel/PCR DNA fragments extraction kit (RBC, Bioscience, Taipei, Taiwan). The purified amplicons were sequenced (Bioneer Corporation, Daejeon, South Korea) before assembly using the BioEdit alignment program (http://www.mbio.ncsu.edu/BioEdit/bioedit.html). Sequences were compared with existing 18S rRNA sequences on GenBank using BLAST searches (http://www.ncbi.nlm.nih.gov/blast/Blast.cgi).

**Table 1:** Specific primers for the 18s rRNA gene of amoebae.

Primer name	Nucleotide sequence (5’ to 3’)
18s-Vahl-1-F	GATCCTGCCAGTAGTCATATGC
18s-Vahl-1-R	CGCTATGTCTTGTCACTACCTC
18s-Vahl-II-F	TTCTGGAGAGAGAGCCTGAG
18s-Vahl-II-R	CTATTGGAGCTGGAATTACCG
18s-Vahl-III-F	ATTGGAGGACAAGTCTGGTG
18s-Vahl-III-R	GACTACGACGGTATCTGATC
18s-Vahl-IV-F	CAGGGACGAAAGTTAAGGGATC
18s-Vahl-IV-R	GCATCACAGACCTGTTATTGCC
18s-Vahl-V-F	ATTGGGTGGTGGTGCATGG
18s-Vahl-V-R	CTAGGAATTCCTCGTTCACG

### Bacterial strains and growth conditions

*B*. *pseudomallei* isolated from the positive soil site, Ban Kai Na in Nam Phong district, Khon Kaen, Thailand [5, 6] was used throughout this study. *B*. *pseudomallei* was previously isolated and identified [5, 31]. In brief, 100 g soil was vigorously mixed with 100 mL distilled water before left for 30 min to allow sedimentation. Thereafter, 500 μL of the supernatant was plated onto modified Ashdown’s agar and incubated at 37°C and visually inspected daily for 4 to 7 days. *B*. *pseudomallei*-suspected colonies (wrinkled or smooth with purple-pink color) were verified by triple sugar iron (TSI), augmentin/colistin susceptibility, assimilation of L-arabinose test, latex agglutination [32] and PCR using the specific primers (BpTT4176F and BpTT4290R) [33]. *B*. *pseudomallei* was stored in Luria Bertani (LB) with 45% glycerol at -80°C.

Bacteria from frozen stocks was streaked on an Ashdown’s agar plate and incubated at 37°C for 48 h. A single colony of *B*. *pseudomallei* was thereafter cultured in 3 mL LB broth at 37°C, 200 rpm, 16 h before being harvested and washed twice at 8,000 ×g, 30 sec and resuspended in PAS to 10^7^ cells/mL for co-culture investigation.

Nonpathogenic *E*. *coli* strain SM-10 was grown in LB broth at 37°C, 200 rpm for 16 h and used as a food source (MOI 100) to maintain amoeba species at the trophozoite stage during cultivation.

### Co-culture of amoeba trophozoites and *B*. *pseudomallei*

To investigate intracellular viability of *B*. *pseudomallei* in amoebae, trophozoites of each amoeba species were harvested by centrifugation at 500×g for 3 min, washed 3 times and resuspended in PAS in a 24-well plate (5x10^3^ cells/well) before being allowed to form a monolayer for 15 min. Subsequently, *B*. *pseudomallei* cells in PAS at the optimal MOI 20 were added to each well containing a monolayer of amoebae and incubated for 1 h at 30°C. Extracellular *B*. *pseudomallei* were removed, first by washing with PAS 3 times then by the kanamycin protection assay at a final concentration of 300 μg/mL kanamycin for 30 min to eliminate extracellular *B*. *pseudomallei*. The complete elimination of extracellular *B*. *pseudomallei* was checked by plate count technique. Afterwards, the culture solution was aspirated by pipette and the remaining monolayer was washed once with PAS and further incubated in PAS at 30°C for another 3 and 6 h. The monolayers of amoebae were then lysed with 0.1% triton-X100 for 20 sec to release internalized *B*. *pseudomallei* [34]. Colony forming units (CFUs) of *B*. *pseudomallei* were enumerated by plating on Ashdown’s agar, the selective media for *B*. *pseudomallei* and incubated at 37°C for 2 days and reported as log10 CFU/mL. All experiments were performed in duplicates, each of three independent experiments.

To examine whether *B*. *pseudomallei* could promote the growth of the amoebae, 1 mL of approximately 10^4^/mL amoeba trophozoites in PAS in a 1.5 mL-microtube were co-cultured with *B*. *pseudomallei* at MOI 100 and incubated at 30°C for 0, 6, 12, 18 and 24 h. In parallel, positive and negative controls consisted of amoebae fed with *E*. *coli* (MOI 100) and amoebae with no *E*. *coli*, respectively. The amoeba cells at each time point were taken and fixed with a final concentration of 1.25% (v/v) glutaraldehyde before being counted using a hemocytometer. Meanwhile, the population of amoebae were also examined and photographed under a Carl Zeiss upright microscope with built-in camera. The investigations were performed in duplicates of three independent experiments.

### Monitoring of intracellular *B*. *pseudomallei* using a confocal laser scanning microscope

*B*. *pseudomallei* was stained with CellTracker^™^ Orange CMTMR (Invitrogen, USA) according to the manufacturer’s instructions. The CellTracker^™^ Orange-*B*. *pseudomallei* were co-cultured with amoebae at MOI 20 at 30°C for 1 h followed by the kanamycin protection assay in a 24-well plate and further incubated for 3 and 6 h. After gently washing 5 times with PAS, 10 μL of the amoebae were taken and dropped on to a coverslip. Subsequently, the amoeba cells were fixed with 1.25% (v/v) glutaraldehyde for 10 min before FITC-ConA (Sigma, USA) at 50 μg/mL final concentration was applied and incubated at room temperature for 20 min. The cover slip was thereafter placed on a glass slide. The internalized *B*. *pseudomallei* were detected by fluorescence under a LSM 800 confocal laser scanning microscope (CLSM) (Zeiss, Germany). The CellTracker^™^ Orange-*B*. *pseudomallei* and the FITC-ConA-amoeba stained cells were excited with 516 and 488 nm lasers, respectively. Image processing was done using ZEN software.

### Statistical analysis

The results of log_10_ CFU and numbers of amoebae are reported as the mean ± standard deviation (SD) from duplicates of three independent experiments. The numbers of bacteria and amoebae at different time intervals were statistically analyzed using ANOVA (analysis of variance). Statistical analyses were performed using GraphPad Prism software, *p* values less than 0.05 were considered to indicate statistical significance.

## Results

### Amoeba species identification

Amoebae from the *B*. *pseudomallei-*positive soil sample were isolated and maintained as monoxenic cultures with *E*. *coli* as their food source. The three amoeba species were *Paravahlkampfia ustiana*, *Acanthamoeba* sp., and *Naegleria pagei*, identified according to their morphologies and 18S rRNA gene sequences. However, the isolate A-ST39-E1 could not be identified because the 18S rRNA did not match any nucleotide sequences in the database.

**Fig 1:**
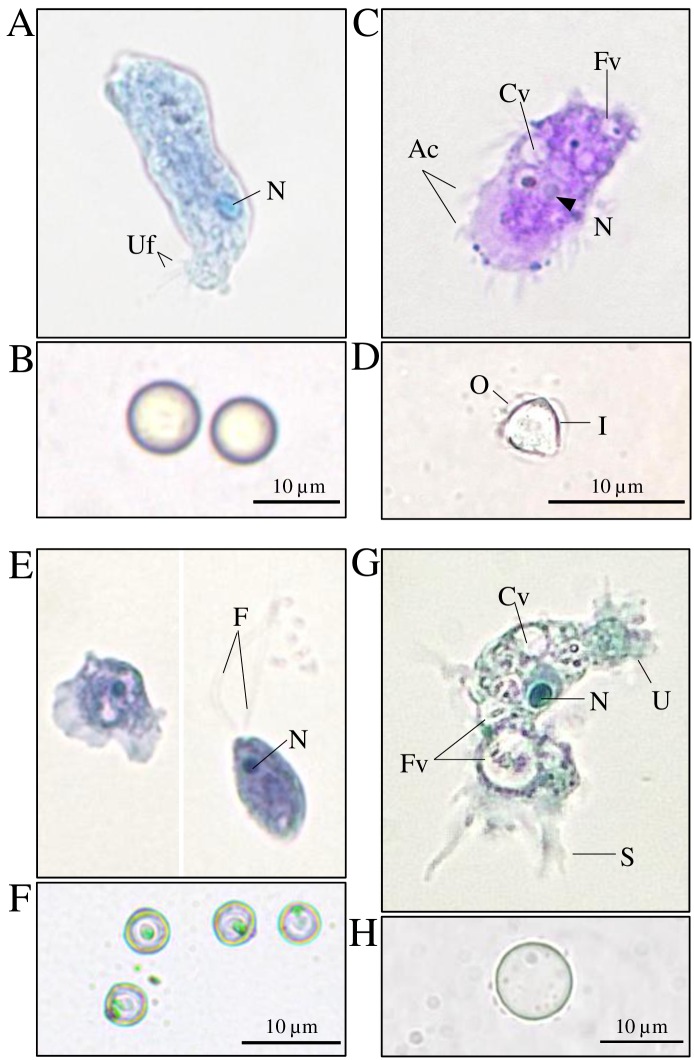
Bright field microscopic images demonstrate trophozoite and cyst morphology (A and B) *Paravahlkampfia ustiana*, (C and D) *Acanthamoeba* sp., (E and F) *Naegleria pagei* and (G and H) isolate A-ST39-E1. *P*. *ustiana*, *N*. *pagei* and isolate A-ST39-E1 were stained with trypan blue while *Acanthamoeba* sp. was stained with crystal violet. The letter in images indicate the following: Uf = Uroidal filaments, N = Nucleus, Ac = Acanthopodia, Cv = Contractile vacuole, Fv = Food vacuole, O = Outer-cyst wall, I = Inner-cyst wall, F = Flagella, S = Sub-pseudopodium and U = Uroid.

The trophozoite of *P*. *ustiana* was elongated and cylindrical (Fig 1A). Cells exhibited eruptive locomotion and possessed a single vesicular nucleus. A rhizoid-like element was present on a part of the cell terminal opposite the direction of movement. The trophozoite was unable to transform into a flagellate state. Cysts were spherical, approximately 8–10 μm in diameter and without pores in the walls (Fig 1B). The 1,692 bp 18S rRNA gene sequence is deposited in the GenBank database (accession number: KX068999). The BLAST result demonstrated 99% similarity to *P*. *ustiana* (GenBank accession number: AJ224890).

*Acanthamoeba* sp. exhibited an amoeboid form without flagellate state, and possessed spine-like structures (acanthopodia) on its surface (Fig 1C). The cyst was star-like with a double-layered wall which is a typical character of the genus *Acanthamoeba* (Fig 1D). The 1,829 bp of the 18S rRNA sequence (GenBank accession number: KX069000) revealed the highest similarity (99% at the nucleotide level) with *Acanthamoeba* sp. (GenBank accession number: AF333608).

*Naegleria pagei* was identified according to the biflagellate and amoeboid stages in its life cycle (Fig 1E). The amoeboid form was typically elongated and possessed laterally-branched pseudopodia. The cyst was spherical, approximately 3–5 μm in diameter, and contained a single granule (Fig 1F). The 1,822 bp 18S rRNA gene sequence (GenBank accession number: KX069001) shared 99% similarity with *N*. *pagei* (GenBank accession number: DQ768721).

Isolate A-ST39-E1; an amoeboid organism, exhibited both trophozoite and cyst stages in its life cycle. The trophozoite was uninucleate with elongated sub-pseudopodia and a bulbous uroid (Fig 1G). Notably, cell shape and size fluctuated among members of a single clone. The cyst was spherical with an average diameter of 10 μm (Fig 1H). The 582 bp partial 18S rRNA gene sequence (GenBank accession number: KX069002) shared no significant similarity with any amoeba species deposited in the GenBank database.

### *Paravahlkampfia ustiana*, *Acanthamoeba* sp. and isolate A-ST39-E1 engulfed and digested *B*. *pseudomallei* cells

We first investigated whether *P*. *ustiana*, *Acanthamoeba* sp., *N*. *pagei* and isolate A-ST39-E1 could engulf and have an impact on the survival of internalized *B*. *pseudomallei* by co-cultivation for 1 h, followed by the kanamycin protection assay to kill extracellular bacteria (considered as 0 h). Meanwhile, we also determined that kanamycin at 300 μg/mL could kill 100% of *B*. *pseudomallei* using bacterial plate count (data not shown).

**Fig 2:**
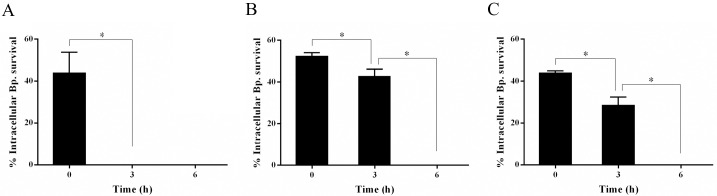
Intracellular survival through time of *B*. *pseudomallei* in *P*. *ustiana* (A), *Acanthamoeba* sp. (B) and isolate A-ST39-E1 (C). Time zero represents 3 hours after *B*. *pseudomallei* feeding. Bars represent the standard errors of the means of duplicate, three times independent experiments, * *p* < 0. 0001 using ANOVA.

**Fig 3:**
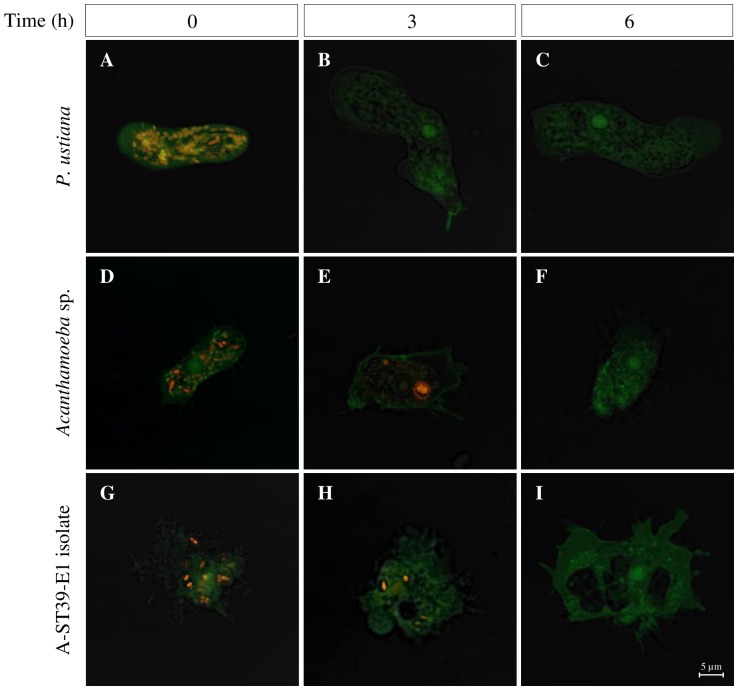
*B*. *pseudomallei* is internalized into amoebae but could not resist digestion. CLSM micrographs show the internalized *B*. *pseudomallei* in *P*. *ustiana* (A-C), *Acanthamoeba* sp. (D-F) and isolate A-ST39-E1 (G-I) at 0, 3 and 6 h after kanamycin treatment. Orange fluorescence represents CellTracker^™^ Orange-*B*. *pseudomallei* and green fluorescence indicates the amoebae stained with FITC-ConA for visualization.

Quantification of colony forming units (CFUs) of the internalized *B*. *pseudomallei* indicated the ability of *P*. *ustiana*, *Acanthamoeba* sp. and isolate A-ST39-E1 to engulf *B*. *pseudomallei* (Figs 2 and 3). On the other hand, *N*. *pagei* could not internalize *B*. *pseudomallei* (data not shown). The percentages of *B*. *pseudomallei* surviving inside the amoebae at 0 h (at the start of the kanamycin protection assay) compared to the number inoculated were 44.17, 52.55 and 44.13 in *P*. *ustiana*, *Acanthamoeba* sp. and isolate A-ST39-E1, respectively (Fig 2). In *P*. *ustiana*, this had dramatically decreased to zero at 3 h (*p* < 0.0001) (Fig 2A). In *Acanthamoeba* sp. and isolate A-ST39-E1, a less dramatic decrease occurred by 3 h (to 42.97% and 28.71%, respectively) (*p* < 0.0001) but no living *B*. *pseudomallei* could be detected inside these amoebae at 6 h (Fig 2B and 2C).

CLSM images revealed that the CellTracker^™^ Orange-*B*. *pseudomallei* were localized intracellularly in vacuoles of *P*. *ustiana*, *Acanthamoeba* sp. and isolate A-ST39-E1 at 0 h after kanamycin treatment (Fig 3A, 3D and 3G). Subsequently, the internalized *B*. *pseudomallei* disappeared within *P*. *ustiana* at 3 and 6 h (Fig 3B and 3C). Reductions in numbers of *B*. *pseudomallei* in the cytoplasm of *Acanthamoeba* sp. and isolate A-ST39-E1 were observed at 3 h (Fig 3E and 3H) and none could be detected at 6 h (Fig 3F and 3I).

These results suggested that *P*. *ustiana*, *Acanthamoeba* sp. and isolate A-ST39-E1, cultured from a *B*. *pseudomallei-*positive soil site, could internalize and digest *B*. *pseudomallei*. During the experimental period, all amoeba cells retained trophozoite appearance: no evidence of cyst formation was seen.

### *Burkholderia pseudomallei* facilitated growth of *Acanthamoeba* sp. and isolate A-ST39-E1

**Fig 4:**
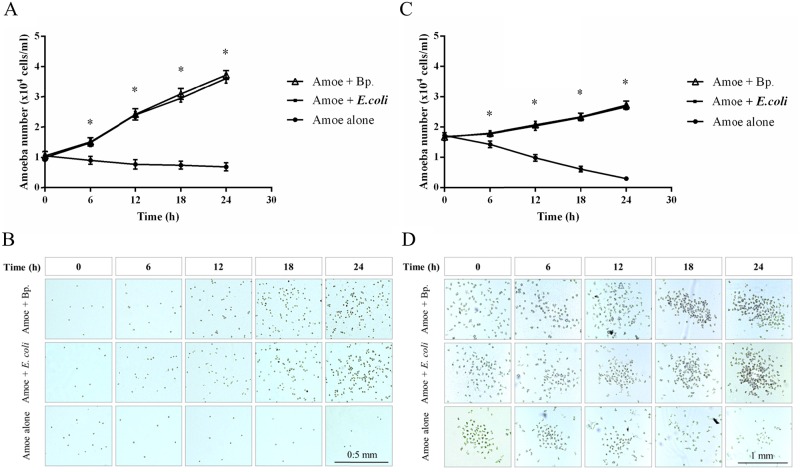
Numbers of *Acanthamoeba* sp. and isolate A-ST39-E1 over time (A-B and C-D respectively) after feeding with *B*. *pseudomallei* (▲) or *E*. *coli* (positive control) (■) or deprived of bacteria as a negative control (●). Graphs and figures show no significant differences between amoebae fed on *B*. *pseudomallei* and *E*. *coli*. However, numbers of amoebae in the negative control group were significantly lower than in the pother groups (*p* ≤ 0.0001). Data are mean ± SD from duplicates of the three independent experiments.

We further observed the numbers of *Acanthamoeba* sp. and isolate A-ST39-E1 co-cultivated with either *B*. *pseudomallei* or *E*. *coli* at MOI 100 at 30°C by direct counting over a 24 h period. Controls were amoebae cultivated without bacterial cells. The results demonstrated that the numbers of both *Acanthamoeba* sp. and isolate A-ST39-E1 increased with time when co-cultured with *B*. *pseudomallei* to an extent comparable to those co-cultured with *E*. *coli*, and significantly higher number than the solo amoebae (*p* < 0.0001) (Fig 4A and 4C). Densities were directly observed under a light microscope (Fig 4B and 4D).

## Discussion

To the best of our knowledge, this is the first time that interactions have been demonstrated between *B*. *pseudomallei* and free-living amoebae isolated from the same soil site of the melioidosis endemic zone. Four species of amoebae, *P*. *ustiana*, *Acanthamoeba* sp., *N*. *pagei* and isolate A-ST39-E1 were taken into monoxenic culture and supplied with living *E*. *coli* as a food source. Our findings, based on a plate count technique and confocal microscopy, revealed that *B*. *pseudomallei* could not survive predation by *P*. *ustiana*, *Acanthamoeba* sp. and isolate A-ST39-E1. Additionally, amoebae could survive and prosper when co-cultured with *B*. *pseudomallei*. Our findings indicate that these three species of amoebae can internalize and digest *B*. *pseudomallei* under the experimental conditions used and therefore do not act as hosts or reservoirs for the bacterium.

The ability of environmental amoebae to graze *B*. *pseudomallei* was previously established by Inglis et al. [17]. They demonstrated that three water-isolated *Acanthamoeba* species, *A*. *astronyxis*, *A*. *castellani*, and *A*. *polyphaga*, could endocytose *B*. *pseudomallei*. Our study has shown that not only *Acanthamoeba* species can graze *B*. *pseudomallei* but also at least another two taxa of soil amoebae, *P*. *ustiana* and isolate A-ST39-E1.

A wide spectrum of interactions between bacteria and environmental protozoa has been demonstrated. Not all bacteria are digested by protozoa grazing on them. Indeed, some pathogenic bacteria evade digestion and can persist in the environment within amoebae [13, 35]. Some remarkable examples of bacterial survival within amoebae have been demonstrated in recent decades, including *Legionella pneumophila* and related species, *Vibrio cholerae*, *Helicobacter pylori*, *Mycobacterium* spp., *Listeria monocytogenes*, *Escherichia coli* O157 and *Pseudomonas aeruginosa* [13]. Amoebae not only provide an ecological niche for those bacteria to persist in the environment but also enhance pathogenicity, rendering them of public health concern [14, 36, 37].

Our findings are consistent with Huws and colleagues [38], who demonstrated predation effects of some common pathogenic bacteria, including *Bacillus cereus*, *Enterococcus faecalis*, Enteropathogenic *E*. *coli* (EPEC), *Salmonella enterica* serovar Typhimurium by the environmental amoeba, *A*. *polyphaga*. However, *Listeria monocytogenes* and methicillin-sensitive *Staphylococcus aureus* (MSSA) were not internalized into *A*. *polyphaga*. Moreover, Akya and colleagues [39] demonstrated the death of *L*. *monocytogenes* cells phagocytosed by *A*. *polyphaga* ACO12 trophozoites within a few hours post-phagocytosis, whereas, *S*. *enterica* serovar Typhimurium C5 cells, used as controls, were able to survive and multiply within *A*. *polyphaga* trophozoites.

Naturally infected *Acanthamoeba* and *Naegleria*, common inhabitants of soil and water that act as evolutionary incubators for *L*. *pneumophila*, have correlated with outbreaks of legionellosis [14, 40, 41]. However, permissive behaviors of amoebae towards *L*. *pneumophila* can vary. Dey and colleagues [42] demonstrated that *Willaertia magna* c2c inhibited the growth of one strain of *Legionella* but not of others belonging to the same serogroup. Conversely, different *L*. *pneumophila* strains inhibited cell growth and induced cell death in *A*. *castellanii*, *Hartmannella vermiformis* and *W*. *magna* Z503 within 3–4 days while *W*. *magna* c2c strain remained unaffected even up to 7 days. The inability of *N*. *pagei* to graze *B*. *pseudomallei* in this study reinforces the food selection behavior previously verified by Xinyao and colleagues [43]. They demonstrated that *Naegleria* sp. strain W2 could consume some filamentous cyanobacteria (e.g., *Anabaena*, *Cylindrospermum*, *Gloeotrichia*, and *Phormidium*) but not *Oscillatoria* and *Aphanizomenon*.

We are aware that our study may not provide a complete insight into the interaction of *B*. *pseudomallei* with protozoa in the same environmental niche. We examined the interaction between *B*. *pseudomallei* and the 4 amoeba species that could be handled in our laboratory. Our experimental scenarios may not represent the real ecological relationships of *B*. *pseudomallei* in the environment. It is likely that the situation in natural soil is more complicated, since a wide variety of microorganisms is present. Furthermore, the physico-chemical nature of soil may be involved in the interactions between protozoan grazers and their prey [44, 45].

In summary, our work has added more information regarding the environmental life-style of *B*. *pseudomallei*. Three of the four species of amoebae isolated in this study could internalize and subsequently digest *B*. *pseudomallei*. The remaining amoeba species had no interaction with *B*. *pseudomallei*. Bacteria and amoebae residing in the same ecosystem near Khon Kaen, Thailand, have a predator-prey relationship. Clearly, not all amoeba species can facilitate the persistence and dispersal of a particular bacterial pathogen in the environment.
